# *Burkholderia* of Plant-Beneficial Group are Symbiotically Associated with Bordered Plant Bugs (Heteroptera: Pyrrhocoroidea: Largidae)

**DOI:** 10.1264/jsme2.ME15153

**Published:** 2015-12-09

**Authors:** Kazutaka Takeshita, Yu Matsuura, Hideomi Itoh, Ronald Navarro, Tomoyuki Hori, Teruo Sone, Yoichi Kamagata, Peter Mergaert, Yoshitomo Kikuchi

**Affiliations:** 1 Graduate School of Agriculture, Hokkaido UniversityKita 9 Nishi 9, Kita-ku, Sapporo 060–8589Japan; 2 Bioproduction Research Institute, National Institute of Advanced Industrial Science and Technology (AIST), Hokkaido Center2–17–2–1 Tsukisamu-higashi, Toyohira-ku, Sapporo 062–8517Japan; 3 Graduate School of Environmental Science, Hokkaido UniversityKita 10 Nishi 5, Kita-ku, Sapporo, 060–0810Japan; 4 Environmental Management Research Institute, National Institute of Advanced Industrial Science and Technology (AIST)16–1 Onogawa, Tsukuba 305–8569Japan; 5 Institute for Integrative Biology of the Cell, Centre National de la Recherche Scientifique (CNRS)avenue de la Terrasse Bât. 34, 91198 Gif-sur-YvetteFrance

**Keywords:** bordered plant bug, gut symbiosis, *Burkholderia*, evolution, symbiont replacement

## Abstract

A number of phytophagous stinkbugs (order Heteroptera: infraorder Pentatomomorpha) harbor symbiotic bacteria in a specific midgut region composed of numerous crypts. Among the five superfamilies of the infraorder Pentatomomorpha, most members of the Coreoidea and Lygaeoidea are associated with a specific group of the genus *Burkholderia*, called the “stinkbug-associated beneficial and environmental (SBE)” group, which is not vertically transmitted, but acquired from the environment every host generation. A recent study reported that, in addition to these two stinkbug groups, the family Largidae of the superfamily Pyrrhocoroidea also possesses a *Burkholderia* symbiont. Despite this recent finding, the phylogenetic position and biological nature of *Burkholderia* associated with Largidae remains unclear. Based on the combined results of fluorescence *in situ* hybridization, cloning analysis, Illumina deep sequencing, and egg inspections by diagnostic PCR, we herein demonstrate that the largid species are consistently associated with the “plant-associated beneficial and environmental (PBE)” group of *Burkholderia*, which are phylogenetically distinct from the SBE group, and that they maintain symbiosis through the environmental acquisition of the bacteria. Since the superfamilies Coreoidea, Lygaeoidea, and Pyrrhocoroidea are monophyletic in the infraorder Pentatomomorpha, it is plausible that the symbiotic association with *Burkholderia* evolved at the common ancestor of the three superfamilies. However, the results of this study strongly suggest that a dynamic transition from the PBE to SBE group, or *vice versa*, occurred in the course of stinkbug evolution.

A number of insects that exclusively feed on a nutritionallypoor diet or indigestible food such as plant sap, vertebrate blood, or woody materials, possess symbiotic microorganisms in their bodies (5, 6, 38). In order to ensure the acquisition of beneficial microbes by their offspring, these insects have evolved sophisticated mechanisms for vertical symbiont transmission, such as ovarial transmission in aphids (50), milk-gland transmission in tsetse flies (3), and coprophagy in termites (29). Recent studies have revealed that several insects, including alydid bugs and allied heteropterans (37, 40), western flower thrips (14), and white flies (10), do not vertically transmit their symbiotic bacteria. Instead, these insects acquire symbiotic microorganisms from the environment every generation. Due to fragmented information for each insect lineage, the evolutionary history of symbiotic associations in species with horizontal transmission still remains unclear.

Among the Insecta, the infraorder Pentatomomorpha of the order Heteroptera may be regarded as an ideal model to elucidate the evolutionary history and mechanisms underlying symbiotic associations with horizontal transmission, since both, microbial symbioses with vertical and horizontal transmission, have evolved in this well-defined, single taxonomic group. The Pentatomomorpha, consisting of more than 12,500 species, is grouped into five superfamilies: Aradoidea, Pentatomoidea, Pyrrhocoroidea, Coreoidea, and Lygaeoidea (25, 62). Except for mycophagous Aradoidea, most members of Pentatomomorpha are phytophagous (40, 60). A number of phytophagous species develop crypts or tubular outgrowths in the midgut posterior region, wherein symbiotic bacteria are housed (6, 24, 51). Members of the superfamily Pentatomoidea possess gammaproteobacterial symbionts and transmit microbes vertically through diverse mechanisms, such as egg smearing, capsule transmission, and jelly transmission (1, 22, 26, 27, 32, 33, 39, 42, 55, 56, 67). On the other hand, most members of the superfamilies Coreoidea and Lygaeoidea are associated with symbionts of a specific clade of the betaproteobacterial *Burkholderia*, called the “stinkbug-associated beneficial and environmental (SBE)” group, which are maintained not by vertical transmission, but by environmental acquisition (23, 37, 40, 53). However, the partial vertical transmission of *Burkholderia* symbionts was recently reported in chinch bugs (4, 31).

In phylogenetic analyses based on morphological and molecular data (25, 28, 73), the relationship between the pentatomomorphan superfamilies has been estimated as: (Aradoidea + (Pentatomoidea + (Pyrrhocoroidea + (Coreoidea + Lygaeoidea)))). On the basis of phylogeny, in conjunction with a broad survey of *Burkholderia* infections, we previously hypothesized that the *Burkholderia* symbiosis evolved from a common ancestor of the superfamilies Coreoidea and Lygaeoidea (40). However, a recent extended survey by Sudakaran *et al.* (65) on the superfamily Pyrrhocoroidea revealed that several species of the pyrrhocoroid family Largidae, commonly known as bordered plant bugs, are associated with *Burkholderia*, which strongly suggests that the evolutionary origin of *Burkholderia* symbiosis is more ancient than we previously estimated (65). This finding suggests that Largidae is the key taxonomic group for elucidating the evolutionary history of *Burkholderia* symbiosis in the infraorder Pentatomomorpha. However, the phylogenetic position and biological nature of the *Burkholderia* symbionts in this family have not yet been investigated in detail.

In the present study, we characterized symbiotic associations in three largid species, *Physopelta gutta* (Fig. 1A), *P. parviceps*, and *P. slanbuschii*, with a focus on the phylogenetic placement of the symbionts. Our results demonstrated that bordered plant bugs closely associated with symbionts of the “plant-associated beneficial and environmental (PBE)” group of the genus *Burkholderia*, which is phylogenetically distinct from the SBE group, highlighting a dynamic transition from the PBE to SBE group, or *vice versa*, that occurred in the course of stinkbug evolution.

## Materials and Methods

### Insects

Insect samples used for cloning and sequencing were collected from different locations in Japan (Table 1) and preserved in acetone until used (21). In order to investigate *Burkholderia* infection in a natural insect population, 24 male and 24 female adults of *P. gutta* collected in Koshi, Kumamoto, Japan, were subjected to diagnostic PCR. Regarding egg inspections, 79 adults of *P. gutta* (38 males, 41 females) collected from the Koshi population were reared in plastic containers (diameter, 184 mm; height, 120 mm) at 25°C under a long-day regimen (16-h light, 8-h dark), fed on sunflower seeds and distilled water containing 0.05% ascorbic acid, and 64 of the oviposited eggs were subsequently subjected to diagnostic PCR.

### DNA extraction

**Fig. 1:**
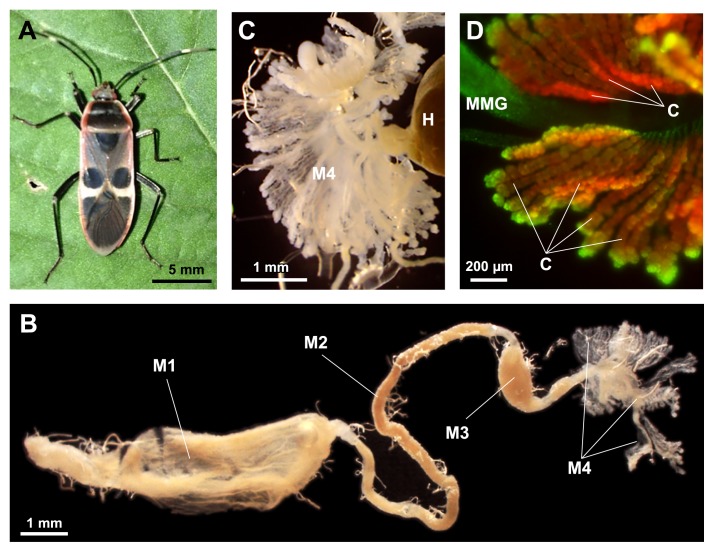
The gut symbiotic organ in *Physopelta gutta*. (A) A female adult of *P. gutta*. (B) The dissected alimentary tract of a female adult of *P. gutta*. (C) An enlarged image of the midgut 4^th^ section (crypt-bearing symbiotic organ). (D) *In vivo* bacterial localization in the midgut 4^th^ section of *P. gutta*, visualized by whole-mount fluorescence *in situ* hybridization with a fluorochrome-labeled *Betaproteobacteria*-specific probe. Red signals indicate betaproteobacterial symbionts, whereas green signals visualize host insect nuclei stained with SYTOX Green. Abbreviations: M1, midgut 1^st^ section; M2, midgut 2^nd^ section; M3, midgut 3^rd^ section; M4, midgut 4^th^ section with tubular outgrowths; H, hindgut; MMG, main duct of the midgut; C, crypts.

Insects were dissected in phosphate-buffered saline (PBS) (137 mM NaCl, 2.7 mM KCl, 8.1 mM Na_2_HPO_4_, 1.5 mM KH_2_PO_4_ [pH 7.4]) with fine forceps and micro-scissors under a dissection microscope, and the midgut fourth section, M4 (crypt-bearing symbiotic organ) (Fig. 1B), was isolated. Total DNA was extracted from the dissected symbiotic organ and eggs using the QIAamp DNA Mini Kit (QIAGEN, Hilden, Germany), according to the manufacturer’s instructions. The quality of extracted DNA was confirmed by NanoDrop 1000 (Thermo Fisher Scientific, Waltham, MA, USA) and also by PCR amplification of the insect cytochrome oxidase I (COI) gene with the primers LCO1490 and HCO2198 (19) (see Table S1).

### Cloning and Sanger sequencing

A 1.5-kb fragment of the bacterial 16S rRNA gene was amplified with the primers 16SA1 and 16SB1 (20) (Table S1). PCR amplification, the cloning of PCR products, and Sanger sequencing of the clones were performed as previously described (36, 48). The sequence reads were assembled with phredPhrap software (17, 18) followed by manual inspections.

### Molecular phylogenetic analyses

Clone sequences were classified into operational taxonomic units (OTUs) by macqiime v1.6.0 (8) and Mothur v1.31.2 (61) based on the furthest-neighbor algorithm with 99% identity threshold. The sequences of clones were subjected to a BLASTN search using the BLAST program (7) against the nucleotide collection (nt) database (downloaded on Feb 2, 2015 from: ftp://ftp.ncbi.nlm.nih.gov/blast/db/). Multiple alignments were constructed by MAFFT v7.032b (L-INS-i) (35) and gap-including and ambiguous sites in the alignments were then removed. Phylogenetic relationships were reconstructed with MEGA 6 (69) under the Tamura-Nei + Γ model of nucleotide substitution (68) based on the maximum likelihood (ML) and neighbor-joining (NJ) methods. The bootstrap values of 1,000 replicates for all internal branches were calculated.

### Whole-mount fluorescence *in situ* hybridization (wFISH)

The dissected alimentary tracts of *P. gutta* were subjected to wFISH, as previously described (31, 45, 49). In order to visualize symbiont localization in the midgut, an Alexa555-labeled, *Betaproteobacteria-*specific oligonucleotide probe BET940 (15) (Table S1) was used. Host nuclei were visualized by SYTOX Green (Invitrogen, Carlsbad, CA, USA). The tissues were observed under a fluorescence microscope (DMI 4000 B, Leica, Wetzlar, Germany). A control assay with no probe was also performed in order to confirm probe-specific fluorescent signals.

### Diagnostic PCR

The primer set 16SA1 and BurkPBE was used for the specific detection of the PBE group of *Burkholderia* (Table S1), targeting a 1.0-kb fragment of the 16S rRNA gene. PCR amplification was performed with BIOTAQ DNA polymerase (BIOLINE, London, UK) and Ampdirect Plus buffer (Shimadzu, Kyoto, Japan) under the following temperature profile: an initial denaturation at 95°C for 10 min, followed by 35 cycles of denaturation at 95°C for 30 s, annealing at 52°C for 1 min, and extension at 72°C for 1 min. The specificity of the primer set was confirmed by checking the PCR amplification of the following target and non-target *Burkholderia* species/strains: target species were *B. phymatum* STM815 and *B. phytofirmans* PsJN (the PBE group of *Burkholderia*), and non-target species/strains were *Burkholderia* sp. strain RPE64, *Burkholderia* sp. strain RPE67, *B. glumae* BGR1, and *B. thailandensis* E264. Specificity was also confirmed by sequencing of the PCR product.

### Quantitative PCR (qPCR)

DNA samples used for cloning and Sanger sequencing were subjected to qPCR of the bacterial 16S rRNA gene in order to estimate the total number of bacteria colonizing the midgut crypts using a LightCycler 96 system (Roche Diagnostics, Basel, Switzerland). A 300-bp fragment of the bacterial 16S rRNA gene was amplified with the universal primer set 515F and 806R (9) (Table S1). The PCR reaction mixture, with a total volume of 20 μL, contained 2.0 μL of 10× PCR buffer (Applied Biosystems, Foster City, CA, USA), 2.0 μL of GeneAmp dNTP Mix (2 mM each of dATP, dTTP, dGTP, and dCTP; Applied Biosystems), 1.2 μL of 25 mM MgCl_2_ solution (Applied Biosystems), 0.2 μL of SYBR Green I (Molecular Probes, Eugene, OR, USA), 0.4 μL of the primer mixture solution (5 μM each of forward and reverse primers), 0.1 μL of AmpliTaq Gold DNA polymerase (Applied Biosystems), 9.1 μL of distilled water, 1.0 μL of dimethyl sulfoxide, and 4 μL of extracted DNA. The PCR temperature profile was 95°C for 10 min, 45 cycles of 95°C for 30 s, 57°C for 30 s and 72°C for 30 s. In order to calculate the absolute number of 16S rRNA gene copies, 10-fold serial dilutions of the target PCR product of *Burkholderia* sp. SFA1 were also amplified, as described previously (31).

### Deep sequencing of the 16S rRNA gene

**Fig. 2:**
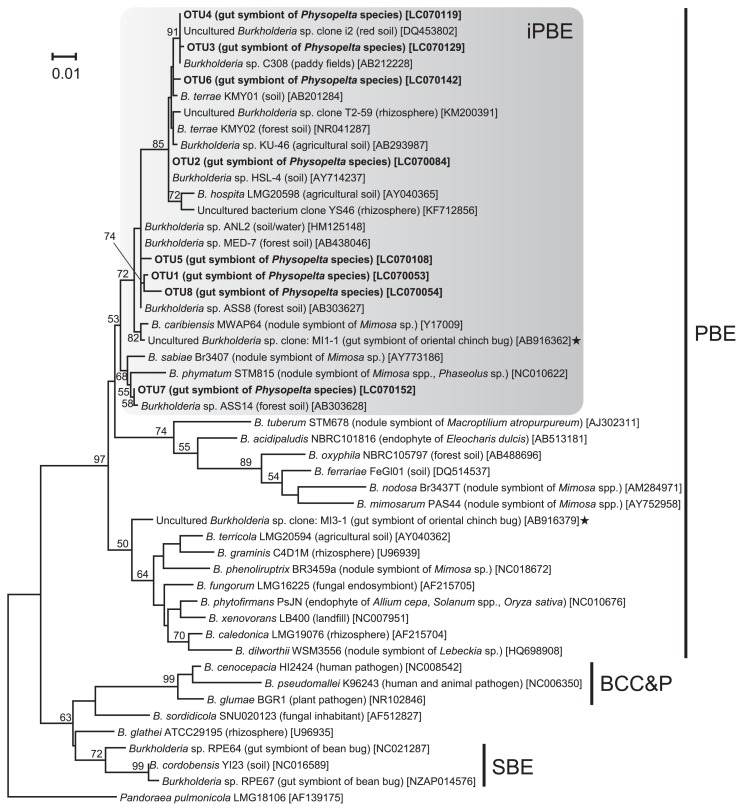
Molecular phylogeny of the gut symbiotic bacteria of *Physopelta* species. The tree displays a maximum likelihood (ML) phylogeny of eight OTUs of the gut symbiotic bacteria identified from *Physopelta gutta*, *P. parviceps and P. slanbuschii* together with selected representatives of the different *Burkholderia* groups. The alignment of 1,356 nucleotide sites of the bacterial 16S rRNA gene was used. The gut symbionts of the *Physopelta* species are shown in bold. The origins or sources of isolation of the *Burkholderia* strains/sequences are represented in parentheses. Accession numbers in the DNA database (DDBJ/EMBL/GenBank) are shown in square brackets. Stars indicate gut symbionts detected from the oriental chinch bug *Cavelerius saccharivorus* (Lygaeoidea: Blissidae) in a previous study (31). The major *Burkholderia* clades (BCC&P, SBE, and PBE) and the subclade “insect-associated PBE (iPBE)” are indicated on the right. Bootstrap values higher than 50% are depicted at the nodes. A phylogeny of all of the 161 *Burkholderia* sequences obtained is shown in Fig. S1. ML and neighbor-joining (NJ) analyses gave essentially the same results (see Fig. S2).

DNA samples used for cloning, Sanger sequencing, and qPCR were subjected to deep sequencing of the 16S rRNA gene. By using a Miseq sequencer (Illumina, San Diego, CA, USA), amplicon sequencing of the V4 region of the bacterial 16S rRNA gene was performed with the universal primers 515F and 806R (9) (Table S1) and analyzed as previously described (31). After joining paired sequences and removing chimeric and low-quality sequences (Q-score<30), the resulting sequences were taxonomically assigned by RDP classifier ver. 2.10.1 (72) with a 50% confident threshold. In the OTU analysis of the *Burkholderia* community, *Burkholderia* sequences were retrieved and clustered into OTUs, which were defined as clusters having <1% sequence differences, using the macqiime program (version 1.6.0) (8). *Burkholderia* clusters (*i.e.*, BCC&P, PBE, and SBE) using the representative sequences of each OTU were identified by a BLASTN search against our collection of *Burkholderia* sequences. These clusters were used to reconstruct the phylogeny shown in Fig. 2.

### Nucleotide sequence accession numbers

The nucleotide sequences of the 16S rRNA genes determined in this study have been deposited in the DDBJ/EMBL/GenBank nucleotide sequence database under the accession no. LC070051 to LC070214. The sequence reads of deep sequencing have been deposited in the DDBJ Sequenced Read Archive under the accession number DRA003821 (see also Table 1).

## Results

### *Physopelta* species form tubular crypts in the M4 section, which house numerous symbiotic bacteria

The midgut of *P. gutta* consisted of four morphologically distinct sections: the stomach-like 1^st^ section (M1), the tubular 2^nd^ section (M2), the swollen 3^rd^ section (M3), and the crypt-bearing 4^th^ section (M4) (Fig. 1B). The midgut crypts appeared to be the tubular-outgrowth type and were arranged in two rows (Fig. 1C). The morphology and arrangement of crypts were similar to those in most members of Lygaeoidea (40), whereas the number of tubular outgrowths appeared to be larger in *P. gutta*. A similar midgut organization and M4 symbiotic organ were observed in *P. parviceps* and *P. slanbuschii*, and there was no sexual dimorphism. These morphological observations were identical to those reported in an early anatomical study by Miyamoto (51).

The dissected midguts of female adults of *P. gutta*, collected in Koshi, Kumamoto, Japan, were subjected to wFISH. Since the midgut microbes were identified as *Burkholderia* species (see below), the *Betaproteobacteria*-specific probe, BET940, targeting 16S rRNA was used to visualize the bacterial residents in the midgut. Bright signals were only detected in the M4 crypts, and not in the main duct of M4 (Fig. 1D). No signals were detected in the other parts of the midgut or in the tissues incubated with no probe (data not shown). This result confirmed that M4 is a symbiotic organ and that the bacteria are localized in its crypts.

DNA samples extracted individually from the midgut crypts of 10 insects of *P. gutta*, four insects of *P. parviceps*, and one insect of *P. slanbuschii*, each representing a different Japanese population (Table 1), were subjected to qPCR in order to quantify the size of the bacterial population per insect. Quantitative PCR revealed that the numbers of copies of the 16S rRNA gene were 8.9×10^6^±1.0×10^7^ (mean±standard deviation [SD], *n*=10) in *P. gutta*, 1.2×10^7^±6.9×10^6^ (mean±SD, *n*=4) in *P. parviceps*, and 2.13×10^7^ (*n*=1) in *P. slanbuschii* (Table 2). These values are similar to those reported for the symbiotic crypts of other stinkbug species (43), and indicate the very dense colonization of *Physopelta* midgut crypts.

### *Physopelta* crypts are nearly exclusively colonized by *Burkholderia*

The 15 DNA samples from the midgut crypts of *P. gutta*, *P. parviceps*, and *P. slanbuschii*, used in the qPCR analysis (Table 1) were subjected to the PCR amplification, cloning, and Sanger sequencing of a 1.5-kb fragment of the bacterial 16S rRNA gene. A total of 164 clones were sequenced and subjected to a BLAST search. The top BLAST hits of all sequences, except for three, were the 16S rRNA gene sequences of *Burkholderia* species. The three exceptional sequences exhibited the highest BLAST hits against either of two alphaproteobacterial species: *Rickettsia bellii* (accession number NR074484) and *Lariskella arthropodarum* (JQ726735). The 161 *Burkholderia* sequences were classified into eight OTUs based on the furthest-neighbor algorithm with a 99% sequence identity threshold (Table 3). The results obtained indicated that (i) all individuals were infected with multiple *Burkholderia* OTUs, except for one individual (*P. parviceps* individual #3 [sample ID, Ppa3]), which was solely infected with *Burkholderia* OTU1, (ii) most of the OTUs were shared by two or three *Physopelta* species, and (iii) OTU1 was the most frequently detected, at 53 out of 161 clones (32.9%); however, OTU1 was not identified in *P. slanbuschii* (Table 3).

### Phylogenetic placement of *Burkholderia* symbionts among plant-associated *Burkholderia*

The genus *Burkholderia*, which is a metabolically and ecologically diverse group in the *Betaproteobacteria*, is grouped into three phylogenetically and ecologically distinct clades: the first clade consists of a large number of human, animal, and plant pathogens, including *B. cepacia*, *B. pseudomallei*, *B. mallei*, and their allied species, designated as the “*B. cepacia* complex and *B. pseudomallei* (BCC&P)” group (13, 59, 63); the second clade includes a number of plant growth-promoting rhizobacteria and nodule-forming plant symbionts, assigned as the PBE group (63); the third clade mainly consists of gut symbionts of the *Coreoidea* and *Lygaeoidea* stinkbugs, assigned as the SBE group (31, 41).

The ML tree of *Burkholderia* OTUs detected from the midgut crypts of the *Physopelta* species is shown in Fig. 2 (see also Fig. S1 showing the phylogeny of all 161 *Burkholderia* sequences identified in this study). All *Burkholderia* detected in the symbiotic organ of *Physopelta* species were placed into the PBE group, in which *Physopelta*-associated *Burkholderia* formed a group with the rhizosphere species *B. hospita* and *B. terrae*, and the nodule-forming legume symbionts *B. caribensis*, *B. phymatum*, and *B. sabiae* (Fig. 2). This PBE subclade, tentatively named here as the “insect- and plant-associated beneficial and environmental (iPBE)” group, was supported by a 53% bootstrap value in the ML analysis. In this subclade, *Physopelta*-associated *Burkholderia* did not form a monophyletic group; instead, they clustered within the group of the rhizosphere species and nodule-forming legume symbionts (Fig. 2). The NJ phylogenetic tree showed a similar result, wherein all *Burkholderia* OTUs detected from the *Physopelta* species belonged to the iPBE clade with a 75% bootstrap value (Fig. S2).

**Fig. 3:**
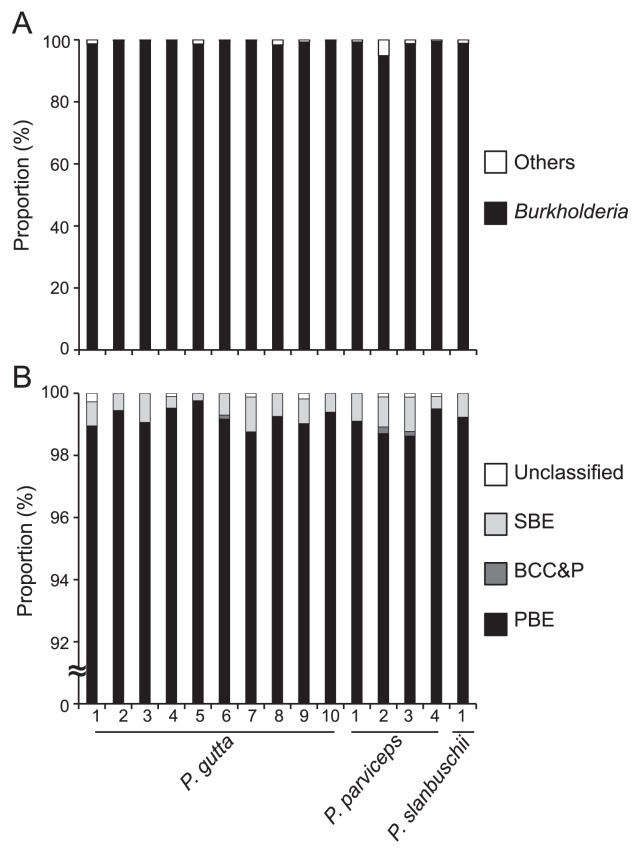
Taxonomic composition of symbiotic microbiota in midgut crypts of *Physopelta* species. (A) Genus level composition. (B) *Burkholderia* clade level composition. The Illumina deep sequences annotated as *Burkholderia* were determined by using a BLASTN search against the reference sequences used in Fig. 2. On the basis of >99% sequence identity, these sequences were categorized into any of the three major *Burkholderia* groups (BCC&P, SBE, or PBE). Note that in all of the individuals of the three *Physopelta* species, sequences of the PBE group of *Burkholderia* accounted for >93% of the total bacterial sequences (see Table 2 for more detailed information).

The 15 insect samples used for qPCR and cloning analyses were subjected to Illumina deep sequencing of the 16S rRNA genes, resulting in 14,794 to 87,816 reads with a median of 26,653 reads for each sample (Table 2). In all 15 insects of the three *Physopelta* species, *Burkholderia* dominantly (>94%) occupied bacterial communities in the midgut crypts (Fig. 3A and Table 2). Further analyses demonstrated that the dominant *Burkholderia* belonged to the PBE group (Fig. 3B and Table 2). These results were consistent with those of the cloning and Sanger sequence analyses. However, since only a 250-bp fragment of the 16S rRNA gene was analyzed by deep sequencing, detailed phylogenetic placements of *Burkholderia*, such as clustering of the iPBE group, were not possible.

In order to further confirm the prevalence of PBE *Burkholderia* in natural populations of the *Physopelta* species, a larger collection of 48 adult individuals of *P. gutta* (24 males and 24 females), collected from Koshi, Kumamoto, Japan, was subjected to the specific PCR detection of the PBE group of *Burkholderia*. All of the 48 insects were positive for infection, indicating the high prevalence of the PBE group of *Burkholderia* in natural populations of *Physopelta* insects.

### Possible horizontal transmission of *Burkholderia* symbionts

In order to clarify the transmission mode of the symbionts, we inspected 64 eggs of *P. gutta* by diagnostic PCR with the PBE-specific primer set. A faint band migrating at the expected size was only detected in two egg samples, while all others were negative (the detection rate was 3.1%: positive/total investigated=2/64). This result strongly suggests that symbionts are rarely transmitted vertically, but are essentially acquired horizontally.

## Discussion

Based on the results of the present study, we conclude that: (i) *Physopelta* species possess a dense population of 10^5^ to 10^7^ cells per insect of symbiotic bacteria in the tubular-type midgut crypts; (ii) symbiotic bacteria belong to the PBE group of the genus *Burkholderia*, wherein the symbionts belong to a specific clade, the iPBE, which includes plant rhizosphere *Burkholderia* species and nodule-forming *Burkholderia* species; (iii) in this clade, *Physopelta*-associated, soil-isolated, rhizosphere and nodule-forming strains do not form coherent groups, but are intermixed; (iv) insects frequently harbor multiple strains/species of *Burkholderia* symbionts, representing host specificity for iPBE species, but a relaxed specificity at the strain level; (v) eggs are rarely infected with *Burkholderia* symbionts, indicating infrequent vertical transmission; and, thus, it is plausible that (vi) largid bugs do not transmit *Burkholderia* symbionts vertically, but acquire them from the ambient environment every generation, as reported for Coreoidea and Lygaeoidea (37, 40). The gut microbiota of other largid species belonging to the genus *Largus*, which was sequenced previously (65), were also dominated by the PBE group of *Burkholderia* (data not shown), suggesting that the Largidae family is consistently associated with this group of *Burkholderia*.

In the infraorder Pentatomomorpha, the superfamilies Pyrrhocoroidea, Coreoidea, and Lygaeoidea form a monophyletic group (25, 28, 73). The Largidae of Pyrrhocoroidea (this work; 65) as well as most members of Coreoidea and Lygaeoidea (23, 37, 40, 53) are associated with environmentally-acquired *Burkholderia*, while members of mycophagous Aradoidea lack gut symbionts (40) and those of Pentatomoidea harbor vertically-transmitted *Gammmaproteobacteria* (1, 22, 26, 27, 32, 33, 39, 42, 55, 56, 67) (Fig. S3). As recently proposed by Sudakaran *et al.* (65), these results strongly suggest that the symbiotic association with environmentally-acquired *Burkholderia* evolved at a common ancestor of the superfamilies Pyrrhocoroidea, Coreoidea, and Lygaeoidea (Fig. S3), and that the midgut crypts as well as *Burkholderia* symbionts were lost in several families including the pyrrhocoroid family Pyrrhocoridae, which is the sister group of Largidae and harbors diverse *Actinobacteria* and *Firmicutes* symbionts in the M3 midgut region (34, 46, 47, 57, 64). Although this hypothesis is plausible, the evolutionary history of *Burkholderia* symbiosis in Pentatomomorpha may be more complex than originally expected. We previously reported that Coreoidea and Lygaeoidea are consistently associated with the SBE group of *Burkholderia* (23, 37, 40, 53) (see also Fig. 2 and S3). On the other hand, the present study revealed that the largid species are closely associated with the PBE group of *Burkholderia*, which is phylogenetically distinct from the SBE group (Fig. 2), indicating that a dynamic transition from the PBE to SBE group of *Burkholderia*, or *vice versa*, occurred during the course of Pentatomomorpha evolution (Fig. S3).

At this stage, the transition history of *Burkholderia* symbionts remains unclear, and there are several evolutionary hypotheses. The high prevalence of the SBE group of *Burkholderia* in two out of the three superfamilies supports an evolutionary scenario in which stinkbugs were initially associated with the SBE group of *Burkholderia* and shifted to the PBE group of *Burkholderia* in the Largidae lineage (Fig. S3). Alternatively, on the grounds that (i) the Pyrrhocoroidea superfamily is phylogenetically placed in the basal branch of the three *Burkholderia*-associated superfamilies (Fig. S3) (28, 73), (ii) members of the family Largidae and superfamily Lygaeoidea commonly develop tubular-type midgut crypts, and (iii) in addition to the largid species, some species of Lygaeoidea are partially associated with the PBE group of *Burkholderia* (Fig. 2) (4, 31), it is possible that stinkbugs were initially associated with the PBE group of *Burkholderia* and were then replaced by the SBE group of *Burkholderia* in the common ancestors of the Coreoidea and Lygaeoidea superfamilies. In addition, it should be also taken into account that both the PBE- and the SBE-group *Burkholderia* were associated with the common ancestor and have been specialized in each stinkbug lineage. Further broad surveys and phylogenetic studies of hosts and symbionts are still needed.

The cohesive phylogenetic pattern of *Physopelta*-associated and plant-associated *Burkholderia* (Fig. 2) implies that members of the iPBE clade have a unique double lifestyle; these *Burkholderia* may be frequently transmitted between insects and plants, and symbiotically associated with both organisms or infect both hosts from the proximate environment. iPBE *Burkholderia*, although speculative, may have adaptations that make them well-suited for symbiotic life with these two very different types of hosts. Lifestyle transitions between these hosts may be possible by the horizontal gene transfer of gene cassettes carrying symbiotic functions specific for one or the other host, *e.g.*, nodulation and nitrogen fixation genes for symbiosis with legumes. Since the *Physopelta* species investigated in the present study mainly feed on *Mallotus* trees (Malpighiales: Euphorbiaceae) in natural fields (2, 30), it is of great interest to inspect the community compositions of *Burkholderia* and their biological functions in *Mallotus* trees, which will lead to a more comprehensive understanding of the lifestyle of the iPBE group of *Burkholderia*.

Previous studies reported that the elimination of symbiotic bacteria in diverse stinkbug species resulted in retarded growth, reduced body size, high mortality, reduced fecundity, and/or abnormal body coloration in host insects (4, 26, 33, 37, 42, 43, 57, 58, 67), indicating that symbiotic bacteria play a pivotal metabolic role in stinkbug hosts. In the *Physopelta* species, the well-developed symbiotic organ (Fig. 1), high symbiont density in the midgut crypts (Table 2), and high prevalence of symbionts in the host population strongly suggest an intimate host-symbiont interaction. In the iPBE group, *B. caribensis*, *B. phymatum*, and *B. sabiae* are known as nodule-forming symbionts in *Mimosa* species, wherein the symbionts fix atmospheric dinitrogen and provide the host with organic nitrogen compounds (11, 12, 16, 70). Many members of the PBE group of *Burkholderia* are recognized as plant growth-promoting rhizobacteria (PGPR) that consume ethylene by ACC (1-aminocyclopropane-1-carboxylic acid) deaminase and promote root elongation (54, 63, 66). Some of the other members of the PBE group are known for their defensive abilities against phytopathogenic fungi (63, 71). These biological abilities may be involved in the symbiotic association with largid insects. The environmentally transmitted nature of largid symbionts implies their cultivability on axenic media; however, we have not yet succeeded in isolating symbionts from the midgut crypts. As demonstrated previously in the bean bug *Riptortus pedestris* (44, 52), cultured symbiotic *Burkholderia* will provide a unique opportunity not only to clarify the biological functions of iPBE symbionts in largid bugs, but also to comprehensively understand the mechanisms responsible for stabilizing stinkbug-*Burkholderia* symbiotic associations.

## Supplementary Material



## Figures and Tables

**Table 1:** Insect samples investigated in this study.

Species Insect ID	Instar	Sex^a^	Collection location (in Japan)	Collection date	Collector	Accession No.	

						Sanger sequencing	Deep sequencing
*Physopelta gutta*							
Pgu1	Adult	F	Iriomote Island, Okinawa	Jul 1, 2009	T. Hosokawa	LC070051–LC070066	DRR042177, DRZ007392
Pgu2	Adult	F	Koshi, Kumamoto	Sep 28, 2009	Y. Kikuchi	LC070067–LC070079	DRR042178, DRZ007393
Pgu3	Adult	F	Tsukuba, Ibaraki	Oct 28, 2009	N. Kaiwa	LC070080–LC070091	DRR042179, DRZ007394
Pgu4	5th	—	Tanoura, Kochi	Sep 16, 2009	Y. Kikuchi	LC070092–LC070103	DRR042180, DRZ007395
Pgu5	Adult	M	Nakamura, Kochi	May 24, 2003	M. Takai	LC070104–LC070113	DRR042181, DRZ007396
Pgu6	Adult	F	Amakusa, Kagoshima	Jun 26, 2009	H. Toju	LC070114–LC070125	DRR042182, DRZ007397
Pgu7	5th	—	Tanegashima Island, Kagoshima	Jul 30, 2009	T. Hosokawa	LC070126–LC070134	DRR042183, DRZ007398
Pgu8	Adult	M	Kirishima, Kagoshima	Jul 8, 2008	H. Toju	LC070135–LC070144	DRR042184, DRZ007399
Pgu9	Adult	F	Ishigaki Island, Okinawa	May 10, 2009	T. Hosokawa	LC070145–LC070151	DRR042185, DRZ007400
Pgu10	5th	—	Tsukuba, Ibaraki	Oct 9, 2008	Y. Kikuchi	LC070152–LC070159	DRR042186, DRZ007401
*P. parviceps*							
Ppa1	Adult	F	Atami, Shizuoka	Jul 12, 2003	T. Hosokawa	LC070160–LC070170	DRR042187, DRZ007402
Ppa2	Adult	M	Kamitonda, Wakayama	Aug 10, 2008	T. Hosokawa	LC070171–LC070181	DRR042188, DRZ007403
Ppa3	Adult	F	Tsuno, Kochi	Jul 13, 2002	M. Takai	LC070182–LC070193	DRR042189, DRZ007404
Ppa4	Adult	M	Koshi, Kumamoto	Sep 22, 2014	Y. Kikuchi	LC070194–LC070204	DRR042190, DRZ007405
*P. slanbuschii*							
Psl1	Adult	F	Ishigaki Island, Okinawa	Jul 14, 2002	K. Kohno	LC070205–LC070214	DRR042191, DRZ007406

a F, female; M, male; —, undetermined.

**Table 2:** Quantitative PCR and deep sequencing of 16S rRNA genes of symbiotic microbiota in midgut crypts.

Insect ID	No. of copies^a^	No. of sequences^b^						

		Bacteria Total	*Burkholderia* Total^c^	*Burkholderia* Total^d^	PBE^d^	BCC&P^d^	SBE^d^	UNC^d^,^e^
*Physopelta gutta*								
Pgu1	1.53×10^7^	26,692	26,339	21,644 (7)	21,417 (3)	0	168 (2)	59 (2)
Pgu2	5.94×10^6^	30,945	30,828	28,104 (4)	27,949 (2)	0	155 (2)	0
Pgu3	3.30×10^7^	32,864	32,830	28,672 (3)	28,404 (1)	0	268 (2)	0
Pgu4	3.27×10^6^	25,564	25,554	21,518 (3)	21,416 (1)	0	79 (1)	23 (1)
Pgu5	5.12×10^5^	19,288	19,020	16,513 (2)	16,474 (1)	0	39 (1)	0
Pgu6	1.23×10^7^	26,653	26,588	24,200 (4)	24,000 (1)	31 (1)	169 (2)	0
Pgu7	1.60×10^6^	26,452	26,420	24,604 (4)	24,299 (1)	0	276 (2)	29 (1)
Pgu8	1.53×10^6^	51,568	50,697	46,290 (3)	45,946 (1)	0	344 (2)	0
Pgu9	1.34×10^7^	16,660	16,540	12,331 (5)	12,211 (1)	0	98 (3)	22 (1)
Pgu10	2.02×10^6^	72,410	72,348	66,471 (4)	66,068 (3)	0	403 (1)	0
*P. parviceps*								
Ppa1	4.28×10^6^	28,933	28,727	26,534 (4)	26,296 (1)	0	238 (3)	0
Ppa2	8.21×10^6^	87,816	83,302	70,236 (6)	69,326 (1)	149 (1)	680 (3)	81 (1)
Ppa3	1.72×10^7^	21,337	21,070	19,494 (6)	19,226 (1)	29 (1)	215 (3)	24 (1)
Ppa4	1.83×10^7^	21,153	21,051	18,835 (3)	18,741 (1)	0	74 (1)	20 (1)
*P. slanbuschii*								
Psl1	2.13×10^7^	14,794	14,623	12,997 (3)	12,897 (1)	0	100 (2)	0

a The number of 16S rRNA gene copies per individual was determined by qPCR.

b The numbers in parentheses indicate the number of OTUs.

c Assignment was performed by the RDP classifier with a 50% confidence threshold.

d The indicated number is only for reproducible OTUs containing >0.1% *Burkholderia* sequences in each library.

e The number of sequences assigned as unclassified *Burkholderia* species.

**Table 3:** Numbers of 16S rRNA gene clones assigned in each OTU of the *Burkholderia*.

Insect ID	*Burkholderia*								Insect total

	OTU1	OTU2	OTU3	OTU4	OTU5	OTU6	OTU7	OTU8	
*Physopelta gutta*									
Pgu1	5	1	4	2			3	1	16
Pgu2	10	3							13
Pgu3	4	6	1		1				12
Pgu4	1	5	4		2				12
Pgu5	3				6				9
Pgu6		4	3	5					12
Pgu7	2		5	2					9
Pgu8	3	2			1	4			10
Pgu9		3	2	2					7
Pgu10	1	1	1				5		8
*P. parviceps*									
Ppa1	3	4		4					11
Ppa2	8				1				9
Ppa3	12								12
Ppa4	1	10							11
*P. slanbuschii*									
Psl1			3	1		6			10
OTU total	53	39	23	16	11	10	8	1	161
