# Supplementary material for: Burkholderia of Plant-Beneficial Group are Symbiotically Associated with Bordered Plant Bugs (Heteroptera: Pyrrhocoroidea: Largidae)
Source: Microbes Environ. 2015 Dec 9;30(4):321–9. doi: 10.1264/jsme2.ME15153 (PMC4676555; doi:10.1264/jsme2.ME15153)
Supplement: Supplementary file 1 [file 30_321_s1.pdf]

Table S1. Primers and a probe used in this study.

Target group	Target gene	Primer/Probe name	Sequence (5' → 3')	Reference[a]
Primers				
Eubacteria	16S rRNA	16SA1	AGAGTTTGATCMTGGCTCAG	(20)
		16SB1	TACGGYTACCTTGTTACGACTT	(20)
Eubacteria	16S rRNA	515F	GTGCCAGCMGCCGCGGTAA	(9)
		806R	GGACTACHVGGGTWTCTAAT	(9)
Burkholderia PBE clade	16S rRNA	16SA1	AGAGTTTGATCMTGGCTCAG	(20)
		BurkPBE	ACCTCTCAGCAAGGTTCCGTA	This study
Invertebrates	*CO1*	LCO1490	GGTCAACAAATCATAAAGATATTGG	(19)
		HCO2198	TAAACTTCAGGGTGACCAAAAAATCA	(19)
Probe				
Betaproteobacteria	16S rRNA	BET940	Alexa555-TTAATCCACATCATCCACCG	(14)

[a] Detailed information of the references:

9. Caporaso, J.G., C.L. Lauber, W.A. Walters, et al. 2012. Ultra-high-throughput microbial community analysis on the Illumina HiSeq and MiSeq platforms. ISME J 6: 1621–1624.

14. Demanèche, S., H. Sanguin, J. Poté, E. Navarro, D. Bernillon, P. Mavingui, W. Wildi, T.M. Vogel, and P. Simonet. 2008. Antibiotic-resistant soil bacteria in transgenic plant fields. Proc Natl Acad Sci U S A 105: 3957–3962.

19. Folmer, O., M. Black, W. Hoeh, R. Lutz, and R. Vrijenhoek. 1994. DNA primers for amplification of mitochondrial cytochrome c oxidase subunit I from diverse metazoan invertebrates. Mol Mar Biol Biotechnol 3: 294–299.

20. Fukatsu, T., and N. Nikoh. 1998. Two intracellular symbiotic bacteria from the mulberry psyllid *Anomoneura mori* (insecta Homoptera). Appl Environ Microbiol 64: 3599–3606.

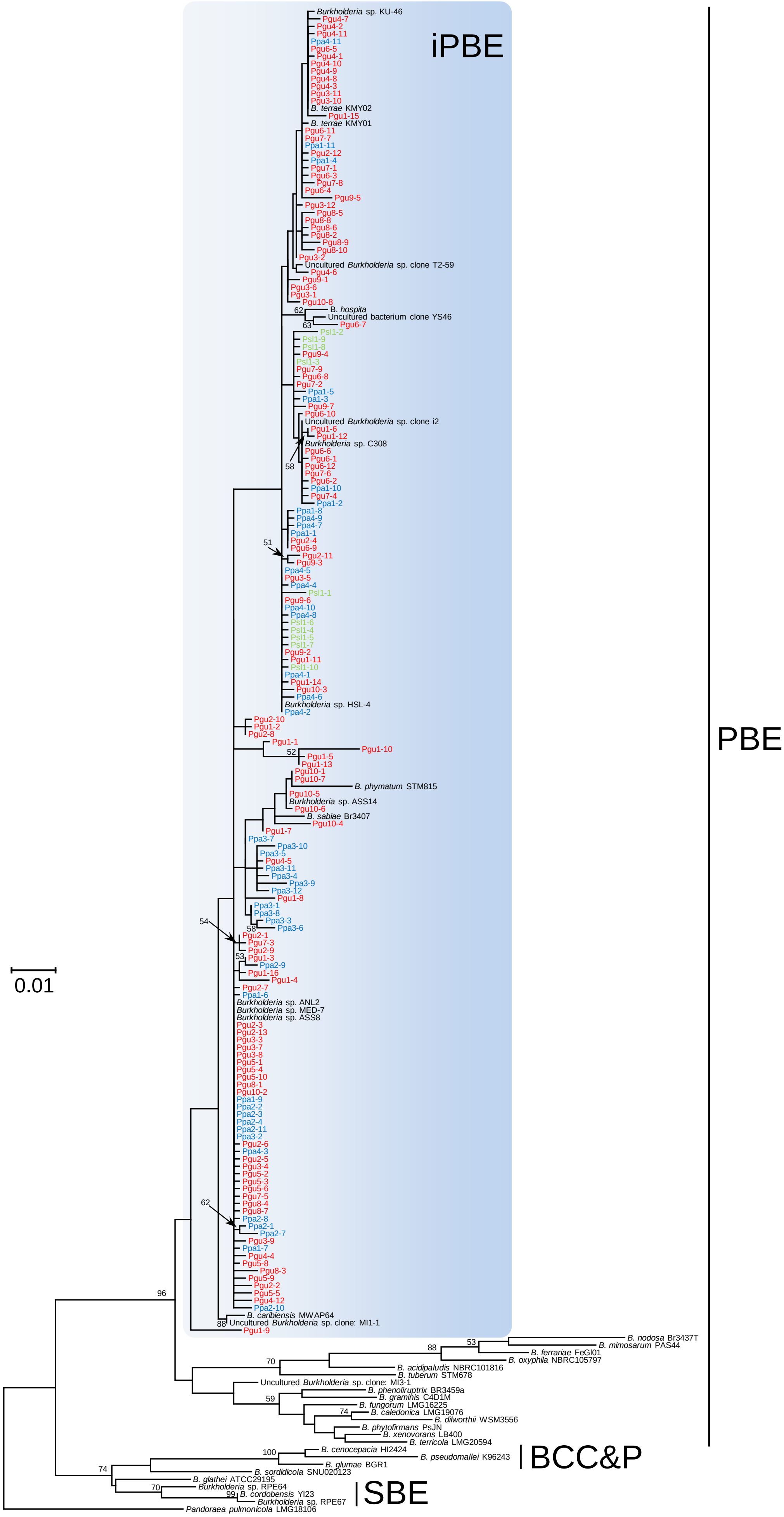

iPBE
PBE
BCC&P
SBE
0.01
Burkholderia sp. KU-46
Pgu4-7
Pgu4-2
Pgu4-11
Ppa4-11
Pgu6-5
Pgu4-1
Pgu4-10
Pgu4-9
Pgu4-8
Pgu4-3
Pgu3-11
Pgu3-10
B. terrae KMY02
Pgu1-15
B. terrae KMY01
Pgu6-11
Pgu7-7
Ppa1-11
Pgu2-12
Ppa1-4
Pgu7-1
Pgu6-3
Pgu7-8
Pgu6-4
Pgu9-5
Pgu3-12
Pgu8-5
Pgu8-8
Pgu8-6
Pgu8-2
Pgu8-9
Pgu8-10
Pgu3-2
Uncultured Burkholderia sp. clone T2-59
Pgu4-6
Pgu9-1
Pgu3-6
Pgu3-1
Pgu10-8
62
B. hospita
Uncultured bacterium clone YS46
63
Pgu6-7
Psl1-2
Psl1-9
Psl1-8
Pgu9-4
Psl1-3
Pgu7-9
Pgu6-8
Pgu7-2
Ppa1-5
Ppa1-3
Pgu9-7
Pgu6-10
Uncultured Burkholderia sp. clone i2
Pgu1-6
Pgu1-12
Burkholderia sp. C308
Pgu6-6
Pgu6-1
Pgu6-12
Pgu7-6
Pgu6-2
Ppa1-10
Pgu7-4
Ppa1-2
58
Ppa1-8
Ppa4-9
Ppa4-7
Ppa1-1
Pgu2-4
Pgu6-9
51
Pgu2-11
Pgu9-3
Ppa4-5
Pgu3-5
Ppa4-4
Psl1-1
Pgu9-6
Ppa4-10
Ppa4-8
Psl1-6
Psl1-4
Psl1-5
Psl1-7
Pgu9-2
Pgu1-11
Psl1-10
Ppa4-1
Pgu1-14
Pgu10-3
Ppa4-6
Burkholderia sp. HSL-4
Ppa4-2
Pgu2-10
Pgu1-2
Pgu2-8
Pgu1-1
52
Pgu1-10
Pgu1-5
Pgu1-13
Pgu10-1
Pgu10-7
B. phymatum STM815
Pgu10-5
Burkholderia sp. ASS14
Pgu10-6
B. sabiae Br3407
Pgu10-4
Pgu1-7
Ppa3-7
Ppa3-10
Ppa3-5
Pgu4-5
Ppa3-11
Ppa3-4
Ppa3-9
Ppa3-12
Pgu1-8
Ppa3-1
Ppa3-8
Ppa3-3
58
Ppa3-6
54
Pgu2-1
Pgu7-3
Pgu2-9
53
Pgu1-3
Ppa2-9
Pgu1-16
Pgu1-4
Pgu2-7
Ppa1-6
Burkholderia sp. ANL2
Burkholderia sp. MED-7
Burkholderia sp. ASS8
Pgu2-3
Pgu2-13
Pgu3-3
Pgu3-7
Pgu3-8
Pgu5-1
Pgu5-4
Pgu5-10
Pgu8-1
Pgu10-2
Ppa1-9
Ppa2-2
Ppa2-3
Ppa2-4
Ppa2-11
Ppa3-2
Pgu2-6
Ppa4-3
Pgu2-5
Pgu3-4
Pgu5-2
Pgu5-3
Pgu5-6
Pgu7-5
Pgu8-4
Pgu8-7
62
Ppa2-8
Ppa2-1
Ppa2-7
Pgu3-9
Ppa1-7
Pgu4-4
Pgu5-8
Pgu8-3
Pgu5-9
Pgu2-2
Pgu5-5
Pgu4-12
Ppa2-10
96
B. caribiensis MWAP64
88
Uncultured Burkholderia sp. clone: MI1-1
Pgu1-9
53
B. nodosa Br3437T
B. mimosarum PAS44
88
B. ferrariae FeGl01
B. oxyphila NBRC105797
70
B. acidipaludis NBRC101816
B. tuberum STM678
Uncultured Burkholderia sp. clone: MI3-1
B. phenoliruptrix BR3459a
B. graminis C4D1M
59
B. fungorum LMG16225
74
B. caledonica LMG19076
B. dilworthii WSM3556
B. phytofirmans PsJN
B. xenovorans LB400
B. terricola LMG20594
100
B. cenocepacia HI2424
B. pseudomallei K96243
B. glumae BGR1
74
B. sordidicola SNU020123
B. glathei ATCC29195
70
Burkholderia sp. RPE64
99
B. cordobensis YI23
Burkholderia sp. RPE67
Pandoraea pulmonicola LMG18106

Fig. S1. Molecular phylogeny of all the gut symbiotic bacteria of *Physopelta* species detected in this study. A maximum likelihood (ML) phylogeny is shown of 161 clones of the gut symbiotic *Burkholderia* identified from *Physopelta gutta*, *P. parviceps,* and *P. slanbuschii* together with selected representatives of the different *Burkholderia* groups. The alignment of 1,350 nucleotide sites of the bacterial 16S rRNA gene was used. The gut symbionts of the *Physopelta* species are indicated with colors: red, *P. gutta*; blue, *P. parviceps*; and green, *P. slanbuschii*. The origins or sources of isolation of the *Burkholderia* strains/sequences and accession numbers in the DNA database (DDBJ/ EMBL/GenBank) are shown in Fig. 2 and Fig. S2. The major *Burkholderia* clades (BCC&P, SBE and PBE) and a subclade “insect-associated PBE (iPBE)” are indicated on the right. Bootstrap values higher than 50% are depicted at the nodes.

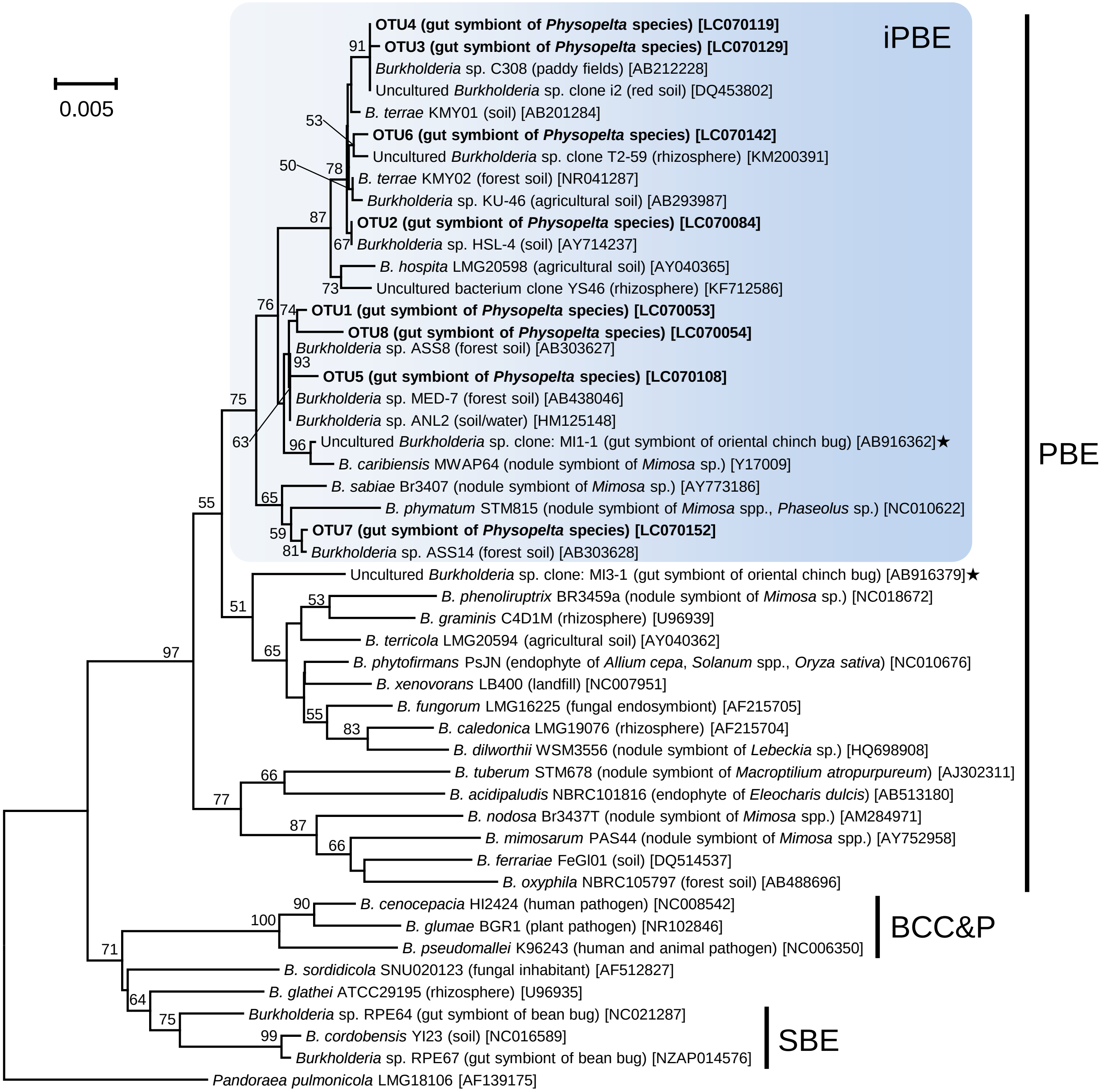

Fig. S2. Molecular phylogeny of the gut symbiotic bacteria of *Physopelta* species. The tree represents a neighbor-joining (NJ) phylogeny of eight OTUs of the gut symbiotic bacteria identified from *Physopelta gutta*, *P. parviceps*, and *P. slanbuschii* together with selected representatives of the different *Burkholderia* groups. The alignment of 1,356 nucleotide sites of the bacterial 16S rRNA gene was used. The gut symbionts of the *Physopelta* species are shown in bold. The origins or sources of isolation of the *Burkholderia* strains/sequences are represented in parentheses. Accession numbers in the DNA database (DDBJ/EMBL/GenBank) are shown in square brackets. Stars indicate gut symbionts detected from the oriental chinch bug *Cavelerius saccharivorus* (Lygaeoidea: Blissidae) in our previous study (31). The major *Burkholderia* clades (BCC&P, SBE and PBE) and a subclade "insect-associated PBE (iPBE)" are indicated on the right. Bootstrap values higher than 50% are depicted at the nodes.

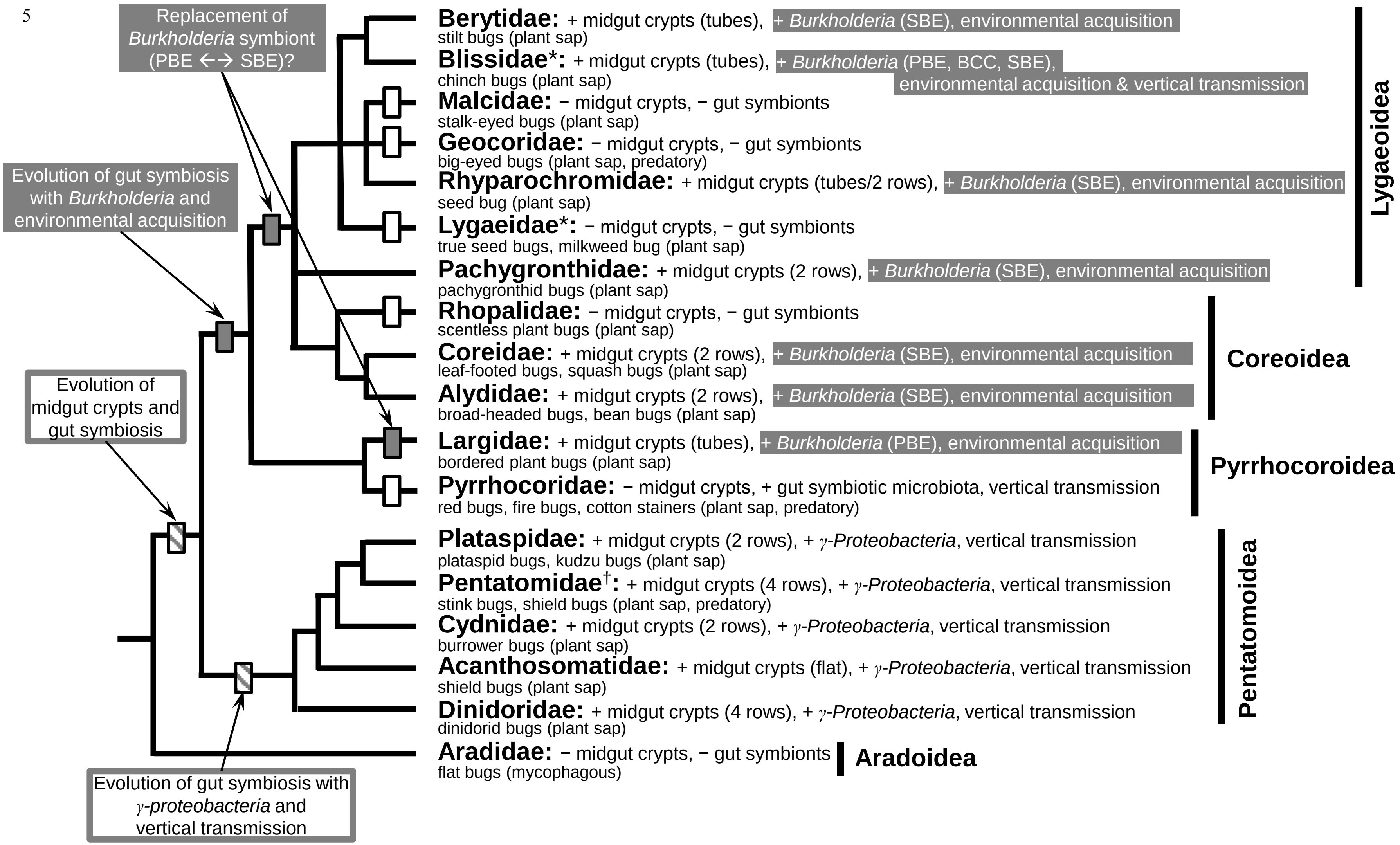

Fig. S3. Evolution of *Burkholderia* symbiosis in the Pentatomomorpha. Phylogeny of higher taxa in the Pentatomomorpha, inferred from previous phylogenetic studies based on molecular data or morphological data (25, 28, 73). Symbiotic systems of the pentatomomorphan families are based on previous studies (1, 4, 6, 22–24, 26, 27, 31–34, 36, 37, 39–42, 46, 47, 49, 51, 53, 55–58, 64, 65, 67). Presence/absence of symbiotic bacteria, symbiont taxonomy, transmission mechanism, and crypt morphology are indicated. Common insect names and their feeding habits are also shown. White rectangles on the phylogeny indicate the loss of the *Burkholderia* symbiosis and midgut crypts. Asterisks (*) indicate the existence of exceptional taxa that have lost the *Burkholderia* symbiosis and midgut crypts and evolved secondarily bacteriocytes carrying endocellular symbionts (46, 47). The dagger (†) indicates the existence of several groups that have lost midgut crypts and gut symbionts (40).
